# The metabolic changes that effect fruit quality during tomato fruit ripening

**DOI:** 10.1186/s43897-022-00024-1

**Published:** 2022-01-20

**Authors:** Feng Zhu, Weiwei Wen, Yunjiang Cheng, Alisdair R. Fernie

**Affiliations:** 1 National R&D Center for Citrus Preservation, Key Laboratory of Horticultural Plant Biology, Ministry of Education, Huazhong Agricultural University, Wuhan, 430070 China; 2 Max-Planck-Institut für Molekulare Pflanzenphysiologie, Am Mühlenberg 1, 14476 Potsdam, Golm Germany

**Keywords:** Tomato, Ripening, Metabolites regulation, Fruit quality

## Abstract

As the most valuable organ of tomato plants, fruit has attracted considerable attention which most focus on its quality formation during the ripening process. A considerable amount of research has reported that fruit quality is affected by metabolic shifts which are under the coordinated regulation of both structural genes and transcriptional regulators. In recent years, with the development of the next generation sequencing, molecular and genetic analysis methods, lots of genes which are involved in the chlorophyll, carotenoid, cell wall, central and secondary metabolism have been identified and confirmed to regulate pigment contents, fruit softening and other aspects of fruit flavor quality. Here, both research concerning the dissection of fruit quality related metabolic changes, the transcriptional and post-translational regulation of these metabolic pathways are reviewed. Furthermore, a weighted gene correlation network analysis of representative genes of fruit quality has been carried out and the potential of the combined application of the gene correlation network analysis, fine-mapping strategies and next generation sequencing to identify novel candidate genes determinants of fruit quality is discussed.

## Introduction

Following import from the Andean region to Europe in the 16th century and human domestication and breeding for around 600 years, tomato has become one of the most economically important vegetables in the world (Bergougnoux 2014). In 2019, the worldwide cultivated area of tomato reached almost 6.11 million hectares producing 243.62 million tons fruits which are sold either as fresh market vegetables or made into soups, juice and ketchup by the processing industry (FAOSTAT, http://www.fao.org/faostat). As the most important traits for fresh market and processing, the appearance and internal quality of fruit are formed by dramatic changes in the activities of a series of metabolic pathways during the ripening process. These metabolic changes are not only attributed to the colorful and flavorsome appearance that were initially required to attract animals to eat and subsequently disperse seeds but also an important nutritional source of carbohydrate, minerals, vitamins, and antioxidants for both animals and humans (Klee and Giovannoni 2011). For this reason, the key biosynthetic pathways of the fruit quality metabolites during the ripening have been well-documented and the genetic and molecular analysis of tomato metabolism have been summarized in several reviews (Carrari and Fernie 2006; Giovannoni 2007; Tohge et al. 2017). Recently, the high quality tomato genome and large scale transcriptomic datasets have significantly accelerated the illumination of the structural genes and transcriptional regulators underlying the formation of high quality fruit (The Tomato Genome Sequencing Consortium 2012). However, given that tomato specialized metabolism is highly complex, in order to identify the novel genes involved in aspects of fruit quality that are associated with these metabolites still requires considerable research effort.

**Fig. 1:**
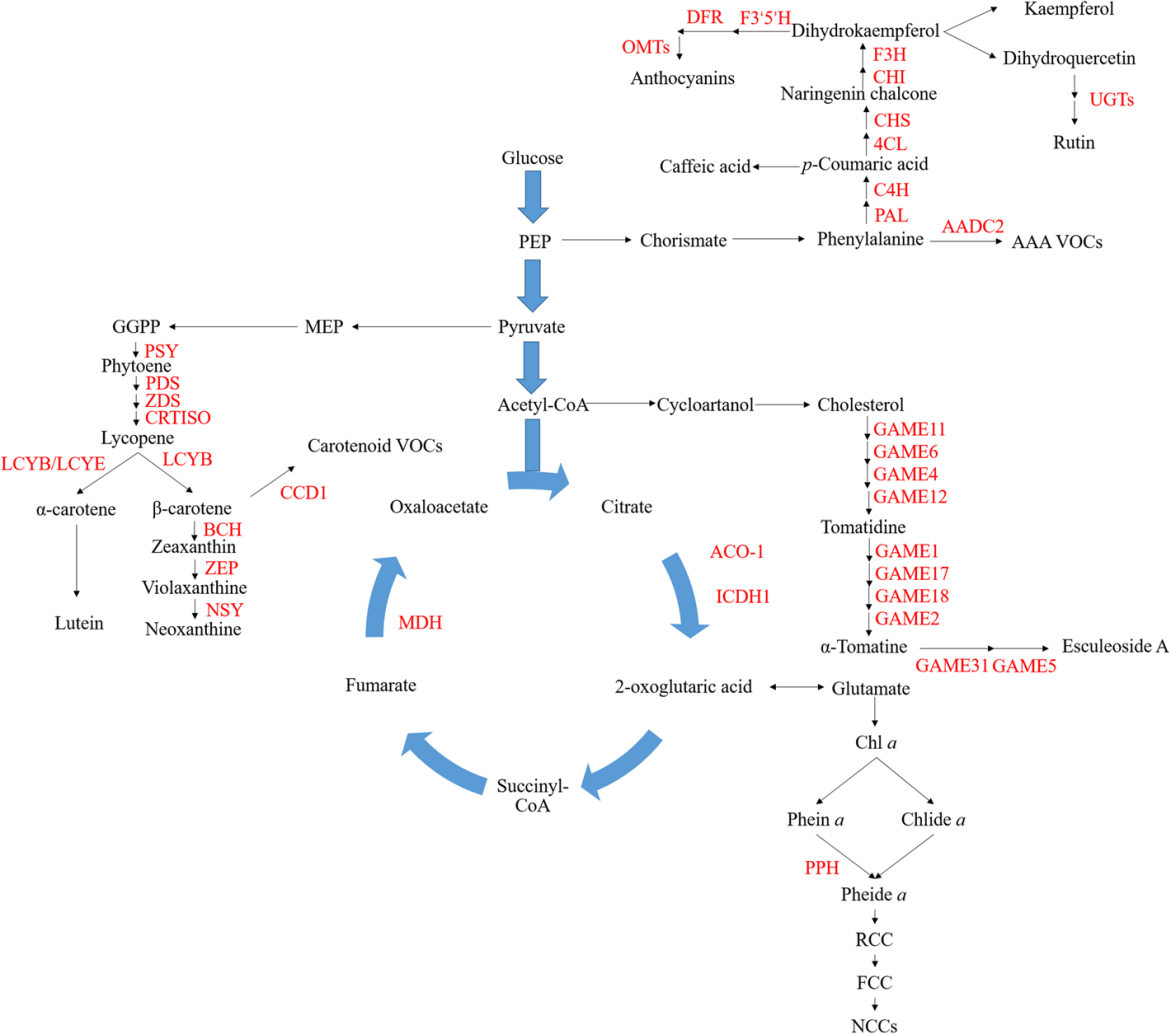
Interrelationships of glycolysis, tricarboxylic acid cycle and fruit quality related metabolism. Names in black letters indicate the metabolite and names in red letters indicate the validated enzymes. VLC Acyl-CoA: Very-long-chain Acyl-CoA; Chl a: Chlorophyll a; Phein a: Pheophytin a; Chlide a: Chlorophyllide a; Pheide a: Pheophorbide a; RCC: Red chlorophyll catabolite; FCC: fluorescent chlorophyll catabolite; NCCs: Nonfluorescent chlorophyll catabolites. ACO-1: Aconitase-1; ICDH1: Isocitrate dehydrogenase 1; MDH: Malate dehydrogenase; PSY, phytoene synthase; PDS, phytoene desaturase; ZDS, ζ-carotene desaturase; CRTISO, carotenoid isomerase; LCYB, lycopene beta cyclase; LCYE, lycopene epsilon cyclase; BCH, β-carotene hydroxylase; ZEP, zeaxanthin epoxidase; NSY, neoxanthin synthase; CCD1, carotenoid cleavage dioxygenase1; PPH: Pheophytin pheophorbide hydrolase; PAL: Phenylalanine ammonia-lyase; C4H: Cinnamate 4-hydroxylase; 4CL: 4-coumarate CoA ligase; CHS: Chalcone synthase; CHI: Chalcone isomerase; F3H: Flavanone 3-hydroxylase; F3'5'H: Flavonoid 3'5'-hydroxylases; DFR: Dihydroflavonol 4-reductase; OMTs: O-methyltransferases; UFGT: UDP glucose flavonoid 3-O-glucosyl transferase; AADC2: Aromatic amino acid decarboxylase 2; GAME: GlycoAlkaloid metabolism

Here, we focus on the recently obtained knowledge considering structural genes as well as transcriptional and post-translational regulators involved in the metabolic pathways underlying both appearance and internal quality such as those involved in chlorophyll, carotenoid, cell wall, central and secondary metabolism (Table 1, Figure 1). Moreover, to mine the other potential genes involved in fruit quality formation, we additionally carried out a weighted gene correlation network analysis of the representative genes of the fruit quality based on previously published high-resolution spatiotemporal transcriptome data for tomato fruit ripening (Shinozaki et al. 2018). We additionally discuss the combined application of gene correlation network analysis, fine-mapping strategies and next generation sequencing as a mean to identify the novel candidate genes underlying fruit quality.

**Table 1:** Validated structure and transcriptional genes of tomato fruit quality metabolism

Metabolite pathway or TF famaily	Gene name	Gene ID	Gene function validation method or regulation pathway	Reference
Chlorophyll degradation	*SGR1*	Solyc08g080090	Map-Based Cloning and transgene	(Barry et al. 2008; Luo et al. 2013)
	*SGRL*	Solyc04g063240	Transgene	(Yang et al. 2020)
	*PPH*	Solyc01g088090	Transgene	(Guyer et al. 2014)
Carotenoid biosynthesis	*PSY1*	Solyc03g031860	Transgene	(Bird et al. 1991)
	*PSY2*	Solyc02g081330	Bacterial mutant complementation	(Bartley and Scolnik 1993)
	*CRTISO*	Solyc10g081650	Map-Based Cloning and *E.coli* transformation	(Isaacson et al. 2002)
	*ZDS*	Solyc01g097810	Transgene	(McQuinn et al. 2020)
	*PDS*	Solyc03g123760	Virus-Induced Gene Silencing (VIGS)	(Naing et al. 2019)
	*LCYE*	Solyc12g008980	*E.coli* transformation	(Roessner-Tunali et al. 2003)
	*LCYB*	Solyc04g040190	Transgene	(Diretto et al. 2020)
	*BCH2*	Solyc03g007960	Map-Based Cloning and *E.coli* transformation	(Galpaz et al. 2006)
	*ZEP*	Solyc02g090890	Map-Based Cloning	(Galpaz et al. 2008)
	*NSY*	Solyc06g074240	*E.coli* transformation	(Bouvier et al. 2000)
	*NXD*	Solyc12g041880	Map-Based Cloning	(Neuman et al. 2014)
Cell wall metabolism	*Exp1*	Solyc06g051800	Transgene	(Brummell et al. 1999)
	*PG*	Solyc10g080210	Transgene	(Jiang et al. 2019)
	*XTHs*	Solyc01g099630	Transgene	(Miedes et al. 2010)
	*PL*	Solyc03g111690	Transgene	(Yang et al. 2017)
	*PE1/PE2*	Solyc03g123630/Solyc07g064170	Transgene	(Wen et al. 2013)
	*TBG4*	Solyc12g008840	Transgene	(Smith et al. 2002)
	*Cel1/Cel2*	Solyc08g081620/ Solyc09g010210	Transgene	(Flors et al. 2007)
	*Xyl1/ Xyl2*	Solyc11g044910/ Solyc01g079570	Transgene and enzyme assay	(Tateishi et al. 2014)
Central metabolism	*Aco-1*	Solyc12g005860	Mutant phenotype analysis	(Carrari et al. 2003)
	*ICDH1*	Solyc01g005560	Transgene	(Gamrasni et al. 2020)
	*MDH*	Solyc07g062650	Transgene	(Centeno et al. 2011)
	*SWEET15*	Solyc09g074530	Transgene	(Ko et al. 2020)
	*SUT1/SUT2*	Solyc11g017010/ Solyc05g007190	Transgene	(Hackel et al. 2006)
	*SWEET 1a*	Solyc04g0646410	Map-Based Cloning and Transgene	(Shammai et al. 2018)
	*LIN5*	Solyc09g010080	Map-Based Cloning and Transgene	(Fridman et al. 2000; Zanor et al. 2009)
	*AgpL1*	Solyc01g109790	Map-Based Cloning and enzyme assay	(Petreikov et al. 2006)
	*VIF*	Solyc12g099190	Transgene	(Qin et al. 2016)
	*INVINH1*	Solyc12g099200	Transgene	(Jin et al. 2009)
	*TIV1*	Solyc03g083910	Transgene	(Klann et al. 1996)
	*SuSy1*	Solyc12g009300	Transgene and enzyme assay	(D'Aoust et al. 1999)
	*TRAMP*	Solyc08g081190	Transgene	(Chen et al. 2001)
	*Frk1/Frk2*	Solyc03g006860/ Solyc06g073190	Transgene	(Odanaka et al. 2002)
	*ALMT9*	Solyc06g072910	GWAS and transgene	(Ye et al. 2017)
Secondary metabolism	*PAL*	Solyc10g086180	Map-Based Cloning and transgene	(Brog et al. 2019)
	*CL*	Solyc08g083110	Map-Based Cloning and transgene	(Brog et al. 2019)
	*C4H*		Transgene	(Millar et al. 2007)
	*4CL*	Solyc12g094520	Introgression line and enzyme activity	(Rigano et al. 2016)
	*CHS1*	Solyc09g091510	Transgene	(Schijlen et al. 2007)
	*CHI*	Solyc05g010320	Map-Based Cloning and transgene	(Kang et al. 2014)
	*F3H*	Solyc02g083860	Map-Based Cloning and transgene	(Maloney et al. 2014)
	*F3’5’H*	Solyc11g066580	Enzyme assay	(Olsen et al. 2010)
	*DFR*	Solyc02g085020	Transgene	(Andrew et al. 1994)
	*CTOMT1*	Solyc10g005060	Transgene and enzyme assay	(Mageroy et al. 2012)
	*AnthOMT*	Solyc06g06450	Transgene	(Gomez Roldan et al. 2014)
	*MOMT1*	Solyc06g083450	Enzyme assay	(Schmidt et al. 2011)
	*MOMT4*		Map-Based Cloning and enzyme assay	(Kim et al. 2014)
	*UGT78-a*	Solyc10g083440	Enzyme assay and transgene	(Tohge et al. 2020)
	*UGTs*	Solyc12g096870 /Solyc12g098600	Map-Based Cloning and transgene	(Alseekh et al. 2020)
	*F3HL*	Solyc03g080190	Transgene	(Meng et al. 2015)
	*FdAT1*	Solyc12g088170	Transgene and enzyme assay	(Tohge et al. 2015)
	*GORKY*	Solyc03g120570	Map-Based Cloning and Transgene	(Kazachkova et al. 2021)
	*GAME31*	Solyc02g062460	Map-Based Cloning and Transgene	(Cardenas et al. 2019)
	*GAME5*	Solyc10g085230	Map-Based Cloning and Transgene	(Szymanski et al. 2020)
	*GAME4/ GAME6/ GAME11/ GAME12/ GAME17/ GAME18/ GAME2*	Solyc12g006460/ Solyc07g043460/Solyc07g043420/Solyc12g006470/ Solyc07g043480/ Solyc07g043500/ Solyc07g043410	Transgene and enzyme assay	(Itkin et al. 2013; Alseekh et al. 2015)
	*GAME1*	Solyc07g043490	Transgene and enzyme assay	(Itkin et al. 2011)
	*SAMT*	Solyc09g091550	QTL mapping and enzyme assay	(Tieman et al. 2010)
	*Lecithin:cholesterol acyltransferase/ LIP1 / LIP2*	Solyc05g050710/ Solyc12g055730/ Solyc03g123750	Map-Based Cloning and Transgene	(Garbowicz et al. 2018)
	*TomLoxC*	Solyc01g006540	pan-genome analysis and Transgene	(Chen et al. 2004; Gao et al. 2019)
	*GAUT10*	Solyc04g064490	GWAS	(Bauchet et al. 2017)
	*PPEAT*	Solyc02g079490	GWAS	(Dominguez et al. 2020)
	*COI1*	Solyc05g052620	Transgene	(Li et al. 2004)
	*AAT1*	Solyc08g005770	Transgene	(Goulet et al. 2015)
	*CCD1A*	Solyc01g087250	Transgene and enzyme assay	(Simkin et al. 2004)
	*CCD1B*	Solyc01g087260	Transgene and enzyme assay	(Ilg et al. 2014)
	*FLORAL4*	Solyc04g063350	Map-Based Cloning and Transgene	(Tikunov et al. 2020)
	*LIP8*	Solyc09g091050	Map-Based Cloning and Transgene	(Li et al. 2020a)
	*AADC1/2*	Solyc08g068610/ Solyc08g006750	Transgene and enzyme assay	(Tieman et al. 2006)
	*ASAT1*	Solyc12g006330	In vitro enzyme assay	(Fan et al. 2016)
	*ASAT2*	Solyc04g012020	In vitro enzyme assay	(Fan et al. 2016)
	*ASAT3*	Solyc11g067270	Map-Based Cloning and Transgene	(Schilmiller et al. 2015)
	*ASAT4*	Solyc01g105580	Map-Based Cloning and Transgene	(Schilmiller et al. 2012)
	*IPMS3*	Solyc08g014230	Map-Based Cloning and enzyme assay	(Ning et al. 2015)
MADS TFs	*CMB1*	Solyc04g005320	Pigmentation	(Zhang et al. 2018a)
	*RIN*	Solyc05g012020	Carotenoid, cell wall and secondary metabolism	(Fujisawa et al. 2013)
	*TDR4*	Solyc06g069430	Secondary metabolism	(Zhao et al. 2019)
	*MBP8*	Solyc12g087830	Cell wall	(Yin et al. 2017)
	*MBP15*	Solyc12g087810	Carotenoid	(Yin et al. 2018)
	*MADS1*	Solyc03g114840	Carotenoid	(Dong et al. 2013)
NAC TFs	*NOR*	Solyc10g006880	Pigmentation and cell wall	(Gao et al. 2020)
	*NOR-like1*	Solyc07g063420	Pigmentation and cell wall	(Gao et al. 2018b)
	*NAC1*	Solyc04g009440	Pigmentation and cell wall	(Ma et al. 2014)
	*NAC4*	Solyc11g017470	Pigmentation	(Zhu et al. 2014)
	*NAP2*	Solyc04g005610	Pigmentation and fruit softening	(Kou et al. 2018; Ma et al. 2018)
MYB TFs	*MYBATV*	Solyc10g086290	Anthocyanin	(Yan et al. 2020b)
	*ANT1*	Solyc10g086260	Anthocyanin	(Schreiber et al. 2012)
	*MIXTA-like*	Solyc02g088190	Primary metabolism	(Ying et al. 2020)
	*MYB72*	Solyc07g055000	Chlorophylls, carotenoids and flavonoids	(Wu et al. 2020)
	*MYB12*	Solyc01g079620	Flavonoid	(Ballester et al. 2010)
	*MYB111*	Solyc06g009710	SGA	(Chen et al. 2019a)
	*AN2*	Solyc10g086250	Anthocyanin and volatile	(Jian et al. 2019; Zhi et al. 2020)
WD40 TFs	*AN11*	Solyc03g097340	Secondary metabolism	(Gao et al. 2018a)
ERF TFs	*ERF.G3-like*	Solyc02g077790	Flavonoid	(Li et al. 2020b)
	*AP2a*	Solyc03g044300	Pigmentation and cell wall	(Karlova et al. 2011)
	*ERF.B3*	Solyc05g052030	Pigmentation	(Liu et al. 2014)
	*GAME9*	Solyc01g090340	GWAS and transgene	(Cardenas et al. 2016; Zhu et al. 2018)
GRAS TFs	*GRAS38*	Solyc07g052960	Pigmentation, secondary metabolism and cell wall	(Shinozaki et al. 2018)
ABF TFs	*AREB1*	Solyc04g078840	Primary metabolic	(Bastias et al. 2014)
ARF TFs	*ARF6A*	Solyc12g006340	Starch and soluble sugars	(Yuan et al. 2019)
	*ARF4*	Solyc11g069190	Cell wall	(Sagar et al. 2013)
	*ARF10*	Solyc11g069500	Sugar accumulation	(Yuan et al. 2018)
BLH TFs	*BL4*	Solyc08g065420	Cell wall metabolism	(Yan et al. 2020a)
bHLH TFs	*GL3*		Anthocyanin	(Nukumizu et al. 2013)
	*TT8*	Solyc09g065100	Anthocyanin	(Qiu et al. 2016)
	*bHLH114*	Solyc01g096370	SGA	(Li et al. 2020b)
	*PRE2*	Solyc02g067380	Pigmentation	(Zhu et al. 2017)
bZIP TFs	*bZIP1*	Solyc01g079480	Amino acid metabolism	(Sagor et al. 2016)
HD-zip TFs	*HZ24*	Solyc04g005800	d-mannose/l-galactose pathway	(Hu et al. 2016)
E3 ubiquitin ligase	*PPSR1*	Solyc01g006810	Carotenoid	(Wang et al. 2020)

### Pigments

As one of the most important traits of fruit appearance quality, pigmentation alters dramatically during fruit ripening process, changing following upregulation of chlorophyll degradation and carotenoid biosynthesis to form the unique color of the fruit (Klee and Giovannoni 2011).

As a representative magnesium porphyrin compound, chlorophylls contain a porphyrin ring chelating a magnesium atom for light energy absorption and the aliphatic hydrocarbon side chain, phytol. The degradation of chlorophylls initializes with the conversion of chlorophyll b to chlorophyll a which is catalyzed by chlorophyll b reductase (Horie et al. 2009). Subsequently, based on the order of removing the phytol and magnesium atoms, the chlorophyll degradation pathway is divided into the PAO (Pheophorbide a monooxygenase) pathway and the PPH (Pheophytin pheophorbide hydrolase) pathway. In the PAO pathway, the phytol group is removed from chlorophyll a which catalyzed by chlorophyllase, and subsequently the magnesium atom chelated with the porphyrin ring is removed as the action of magnesium ion dechelating enzyme. For the PPH pathway, the magnesium atom in the chlorophyll a porphyrin ring is removed first, and then PPH specifically removes the phytol chain of Mg-free chlorophyll (Chl) pigment pheophytin to generate pheophorbide (Schelbert et al. 2009). Then, oxygen atoms are added to the C4 and C5 of the porphyrin ring to break the structure of the porphyrin ring and produce red chlorophyll metabolites (Pruzinska et al. 2003). Subsequently, the red chlorophyll metabolites are converted into a primary fluorescent chlorophyll catabolite and transported out of the chloroplast (Pruzinska et al. 2007). Following modification in the cytosol, these molecules are transported to the vacuole, and finally undergo an isomerization reaction to form the final product of Chl breakdown, nonfluorescent Chl catabolites (Berghold et al. 2004). In addition to the above enzymes, former research has reported that SGR (STAY-GREEN) proteins can interact with chlorophyll degrading enzymes to affect the degradation of chlorophyll. In tomato fruits, SGR1 and SGRL proteins can promote chlorophyll degradation, while the SGR2 protein in Arabidopsis acts as a repressor of the chlorophyll degradation (Barry et al. 2008; Sakuraba et al. 2012; Sakuraba et al. 2014; Yang et al. 2020).

As the main pigment of ripe tomato fruit, the orderly synthesis of carotenoids is a key step of fruit color quality. During the fruit ripening process, carotenoids are *de novo* synthesized by the polymerization of isopentenyl diphosphate to produce geranyl geranyl pyrophosphate (GGPP). GGPP then acts as the direct precursor for synthesis of various linear and epoxidized carotenoids under the catalysis of a series of enzymes. The first reaction which is catalyzed by phytoene synthase (PSY) synthesizes the colorless phytoene from two molecules of GGPP. This enzyme is the key rate-limiting step of carotenoid synthesis pathway and its loss-of-function underlies the *yellow-fruited tomato 2* mutant (Bird et al. 1991; Bartley and Scolnik 1993; Chen et al. 2019b). Subsequently, under the catalysis of phytoene dehydrogenase (PDS), ζ-carotene dehydrogenase (ZDS), ζ-carotene isomerase (Z-ISO) and carotene isomerase (CRTISO), phytoene undergoes dehydrogenation and isomerization reactions to form lycopene, which is the dominate carotenoid of tomato fruit (Hirschberg 2001; Isaacson et al. 2002; Cazzonelli and Pogson 2010). Thereafter, lycopene is converted by lycopene epsilon cyclase (LCYE) and lycopene beta cyclase (LCYB) in the branch pathway of carotenoid synthesis to produce α-carotene and β-carotene (Ronen et al. 1999; Diretto et al. 2020). Moreover, α-, β-carotene can be catalyzed by β-carotene hydroxylase (BCH, loss-of-function which leads to tomato *white-flower* mutant) and through the intermediate products zeinoxanthin and β-cryptoxanthin form lutein and zeaxanthin (Galpaz et al. 2006; Stigliani et al. 2011). In addition, zeaxanthin can also generate antherxanthin and violaxanthin following the reaction catalyzed by zeaxanthin epoxidase (ZEP, loss-of-function which leads to tomato *high-pigment 3* mutant) (Galpaz et al. 2008; Karniel et al. 2020). Finally, violaxanthin can also be converted to neoxanthin under the catalysis of neoxanthin synthase (NSY) (Neuman et al. 2014).

### Cell wall

As one of the predominant parameters of fruit texture and the major determinant of shelf life and commercial value of fruits, cell wall remodeling during the ripening stage is a complex process which contains the hydrolysis of cellulose and hemicelluloses, solubilisation and depolymerisation of the pectin polysaccharides, and rearrangements of their connection (Goulao and Oliveira 2008).

In the cell wall, cellulose is generally cross-linked together with hemicellulose whilst the pectin fills in the spaces of the networks. The hydrolysis of cellulose and hemicelluloses is often catalyzed by cellulase and xyloglucan-endotransglycosylase which can hydrolyze internal 1→4 β-D-glucan linkages. However, suppression of *cellulase* gene expression by antisense method did not alter the tomato fruit softening process, which indicates that they are not the predominant enzymes regulating the cell wall remodeling (Payasi et al. 2009).

As the major components of primary cell wall and middle lamella, pectins are modified with methyl ester groups and highly branched with side-chains of galactosyl and arabinosyl residues in unripe fruits. On ripening initiation, the methyl ester groups and branched side-chains are first removed by pectin methyl esterase (PME), rhamnogalacturonase (RG) and β-galactanase (Wen et al. 2020). Then polygalacturonase (PG) can recognize and hydrolyze the α-1,4-galacturonosyl linkages between galacturonide residues of the de-esterified pectin to produce galacturonide oligomers (Smith et al. 2002; Miedes et al. 2010; Wen et al. 2013; Tateishi et al. 2014; Jiang et al. 2019). However, similar to the result of *cellulase* genes, inhibition of PG activity or antisense of *PME* genes had only minor effects on fruit softening (Smith et al. 1990; Wen et al. 2013). As the key enzyme which breaks the α-1,4-galacturonosyl linkages through β-elimination reaction, silencing *pectate lyase (PL)* dramatically altered the softening process of tomato fruit, emphasizing the vital function of PL for pectin depolymerization in the fruit softening process (Yang et al. 2017).

In addition to the enzymes mentioned above which directly modify cell wall components, expansins are located at the cell wall and are involved in fruit softening by disrupting hydrogen bonds between cellulose microfibrils and xyloglucans (Brummell et al. 1999; Whitney et al. 2000; Perini et al. 2017).

### Central carbon metabolites

As the key components that influence the favor and quality of fruit, central carbon metabolites not only directly affect the sour-sweet taste but also act as important carbon skeletons for other metabolites (Malundo et al. 1995). During fruit ripening process, the content of sugars and organic acids are under highly coordinated regulation of balance importation from source organ as well as utilization via the glycolysis, and the tricarboxylic acid (TCA) cycle (Carrari et al. 2006).

Although the chloroplasts of green fruit can assimilate CO_2_, the majority of fruit photoassimilate is imported from the leaves (Fernie et al. 2020). In leaves, CO_2_ is initially fixed to produce triose phosphates in the chloroplast prior to export to the cytosol to support sucrose biosynthesis. Then sucrose acts as the carbon transportation component to load on the phloem and transported in the sieve for a long distance (Chen et al. 2012). After arriving at the fruit, sucrose is unloaded from the phloem and transported to fruit through two cytological pathways (the apoplastic and symplastic pathway) and stored in vacuoles. In past decades, based on the map-based cloning, several SWEETs (Sugars Will Eventually be Exported Transporters) (*SWEET 1a* and *SWEET15*) and sugar transporters (*SUT1, SUT2* and *SUT4*) involved in sucrose transportation have been cloned and validated to regulate sugar metabolism (Weise et al. 2000; Hackel et al. 2006; Shammai et al. 2018; Ko et al. 2020).

Sucrose metabolism is a key factor in sugar accumulation under the regulation of sucrose-phosphate synthase (SPS), sucrose synthase (SS) and invertase (Ivr). SPS can catalyze uridine diphosphate glucose (UDPG) and fructose 6-phosphate to synthesize sucrose 6-phosphate which subsequently hydrolyzed by sucrose phosphate phosphatase (SPP) to produce sucrose (Dali et al. 1992). Antisense of tomato fruit *sucrose synthase1* (*SuSy1*) not only reduced the sucrose unloading capacity but also affected starch accumulation and fruit development (D'Aoust et al. 1999). According to their subcellular location, Ivrs are divided into apoplastic invertase, cytosolic invertase and vacuolar invertases. Invertase irreversibly catalyzes the degradation of sucrose into glucose and fructose and the well-known QTL (Brix9-2-5) which associated with the glucose and fructose contents is results from the nucleotide polymorphism of apoplastic invertase, *Lin5* in the population (Fridman et al. 2000). Moreover, invertase inhibitors which can bind to invertases and form inactive complexes can also affect the sugar metabolism (Qin et al. 2016).

Unlike sugar metabolites, the organic acids accumulated in the fruit mainly depend on the *de-novo* synthesis in fruit cell. During fruit cell division phase, organic acids are highly accumulated in parallel with the accumulation of soluble sugars (Beauvoit et al. 2014). Subsequently, during the tomato ripening process, respiration is highly induced and organic acids are gradually decreased as the respiratory substrate and then achieve a palatable sugar/acid ratio for consumer (Gautier et al. 2008).

As the two main organic acids in fruits, citric acid and malic acid are the intermediate products of the tricarboxylic acid cycle and phosphoenolpyruvate carboxylase (PEPC) is the key enzyme in organic acid biosynthesis (Carrari et al. 2003; Guillet et al. 2012). The product of glycolysis, PEP is catalyzed by PEPC to form oxaloacetate (OAA). OAA then catalyzed by citrate synthase (CS) and combined with acetyl-CoA to produce citric acid. Moreover, OAA can also reversibly catalyzed by malate dehydrogenase (MDH) to generate malic acid (Centeno et al. 2011). Besides the enzyme of central metabolism, based on a metabolite-based genome-wide association study and BSA mapping, Ye et al. (2017) found that *Al-ACTIVATED MALATE TRANSPORTER9* (*ALMT9* in tomato) is the causal gene of

*TFM6* (the malate content major QTL) and a 3-bp indel in the promoter region of *ALMT9* which destroys a W-box binding site and blocks the regulation of transcription repressor WRKY42 cause the variation of *ALMT9* expression and is attributed to the malate variation among the population.

### Secondary metabolism

The secondary metabolism of tomato fruits can be divided into polyphenols, volatile organic compounds (VOCs) and alkaloids, which act as the bioactive compounds against inflammation, cardiovascular diseases, and cancer (Andersen and Markham 2005).

As the most important component class of the polyphenols, flavonoid metabolites are derived from phenylalanine and synthesized via the phenylpropanoid and polyketide pathways (Perez de Souza et al. 2019). In phenylpropanoid pathway, phenylalanine ammonia lyase (PAL), cinnamate 4-hydroxylase (C4H) and 4-coumaroyl CoA-Ligase (4CL) catalyze the conversion of phenylalanine to 4-coumaronyl-CoA (Millar et al. 2007; Tohge et al. 2014). Subsequently, condensing with three molecules of malonyl-CoA, 4-coumaronyl-CoA catalyzed by chalcone synthase (CHS) produces naringenin chalcone (Schijlen et al. 2007). Then naringenin chalcone is isomerized by chalcone isomerase (CHI) to produce naringenin (Schijlen et al. 2007). Moreover, naringenin is subsequently hydroxylated at position C-3 to form the dihydrokaempferol (DHK) by flavanone-3-hydroxylase (F3H) and then enters different branch metabolism of flavonol and anthocyanin (Tohge et al. 2017).

In the flavonol branch pathway, DHK can be further hydroxylated at the 3′ position to produce dihydroquercetin (DHQ), catalyzed by the P450 hydroxylase, flavonoid 3′-hydroxylase (F3′H). Subsequently, under the control of flavonol synthase (FLS) enzyme, DHK and DHQ are converted to kaempferol and quercetin, respectively (Colliver et al. 2002). Moreover, flavonols can be converted by the flavonoid-3-*O*-glycosyltransferase (F3GlcT) to the representative tomato flavonol-glycosides, such as quercetin 3-*O*-glucoside and quercetin 3-*O*-rutinoside (rutin) (Tohge et al. 2020).

DHK can also be hydroxylated at both the 3′ and 5′ positions by flavonoid 3′, 5′-hydroxylase (F3′5′H) to produce dihydromyricetin (DHM). Under the action of dihydroxyflavonol reductase (DFR) and anthocyanin synthase (ANS), DHM can be catalyzed to anthocyanins (Tohge et al. 2017). Moreover, given that glycosylation is essential for stability, the anthocyanins generally can be modified by glycosylation to produce the most abundant tomato anthocyanins, nasunin and petanin under the regulation of flavonoid glycosyltransferase (UFGT) (such as anthocyanin-3-*O*-glucosyltransferase and anthocyanin-5-*O*-glucosyltransferase) (Tohge et al. 2015). Although anthocyanin is not naturally produced in the cultivar tomato fruit due to the switch off this sub-pathway gene expression in the fruit peel, which may be under the domestication preference of lycopene red color (Gonzali et al. 2009), the bio-fortified tomato which are expressed two transcription factors Delila (*Del*) and Rosea1 (*Ros1*) from snapdragon can significantly induce anthocyanin-related gene expression and accumulate high amount of anthocyanin (Butelli et al. 2008).

VOCs are the important characteristic quality index of fruit, which is mainly composed of a complex mixture of terpenes, aldehydes, alcohols, esters and ketones and other volatile components. Based on the different precursors, VOCs can be divided into four subclasses, (i) fatty acid volatiles, (ii) amino acid derived volatiles, (iii) terpenoid volatiles, and (iv) volatiles derived from carotenoids (Vogel et al. 2010; Klee and Giovannoni 2011).

The formation of fatty acid volatiles, such as trans-2-pentenal and *cis*-3-hexanal are based on lipoxygenase (LOX) oxidation and β-oxidation pathway of fatty acid (Chen et al. 2004). The LOX enzyme catalyzes unsaturated fatty acid to hydroperoxide and then to aldehydes and esters substance under the regulation of hydroperoxide lyase (HPL), alcohol dehydrogenase and alcohol acyltransferase (AAT). Moreover, fatty acid can be catalyzed to acetic acid, butyric acid and caproic acid in β-oxidation reaction, and then deoxygenize to alcohols and then synthesize esters under the action of AAT, whose activity is attribute to the difference of the ester volatiles content of the tomato fruits (*Solanum lycopersicum*) and its closely related species *S. pennellii* (Goulet et al. 2015). Moreover, some lipases which can cleave fatty acids from the glycerol backbone of acylglycerols significantly affect the fatty acid-derived volatile levels (Garbowicz et al. 2018). Recently, Li et al. (2020a) have identified that *LIP8* is highly associated with accumulation of short-chain fatty acid-VOCs (C5 and C6) in tomato fruit by the metabolite-based genome-wide association study. The enzyme assay confirmed that LIP8 can cleave 18:2 and 18:3 acyl groups from glycerolipids and several fruit short-chain fatty acid-VOCs are significantly decreased in *LIP8* CRISPR-edited mutant.

Amino acid derived volatiles mainly use branched chain amino acids (BCAAs) and aromatic amino acids as precursors to synthesize branched chain and phenylpropanoid volatiles. In tomato fruit, α-keto acid intermediate in BCAAs catabolism is the direct precursors for the branched chain flavor volatiles and the glycoconjugation reaction plays an important role in the emission of phenylpropanoid volatiles from ripening tomato fruit (Tikunov et al. 2010; Kochevenko et al. 2012). Moreover, AADC2 (aromatic amino acid decarboxylases 2) and FLORAL4 (a 3-methyl-2-oxobutanoate dehydrogenase) has been confirmed acting as an important regulator of phenylalanine-derived volatiles such as 2-phenylethanol, phenylacetaldehyde and 1-nitro-2-phenylethane (Tieman et al. 2006; Tikunov et al. 2020).

Terpenoid volatiles are synthesized from the condensation of two C5 components, isopentenyl diphosphate and dimethyl allyl diphosphate (Abbas et al. 2017). Based on the carbon skeletons and chemical structure, terpenoid volatiles are divided into isoprene-, monoterpene- and sesquiterpene- derived volatiles and all of them share the common core biosynthesis pathway in plant. To increase the terpenoid volatiles content, the heterologous expressed *S*-linalool synthase (*LIS*) gene of *Clarkia breweri* was found to significantly induce the accumulation of monoterpenes compared to control tomato fruits (Lewinsohn et al. 2001). Moreover, modified the early plastidial terpenoid pathway by expressing the *Ocimum basilicum* geraniol synthase gene can significantly induced the monoterpene accumulation (Davidovich-Rikanati et al. 2007). Recently, the biochemical and *in silicon* analysis has identified the 34 terpene synthase (TPS) genes in tomato genome which contain one isoprene synthase, 10 monoterpene synthases, 17 sesquiterpene synthases and six diterpene synthases as the results of expansions in each clade of the TPS gene family (Zhou and Pichersky 2020).

Besides the important functions as colorants and nutrients, carotenoids also act as the vital precursors for important volatile flavor compounds, such as β-ionone and pseudoionone (Vogel et al. 2010). The production of carotenoid-derived volatiles occurring the non-enzymatic oxidative cleavage of various linear and cyclic carotenoids or by the cleavage action of carotenoid dioxygenase. In tomato, two carotenoid cleavage dioxygenase 1 enzymes (CCD1A and CCD1B) showed differences in their activity towards different substrates and in their double bond preferences. Among them, CCD1B has a more relaxed enzyme specificity which can cleave the C9′–C10′, C13–C14 and C11′–C12′ double bonds of 9-*cis*-β-carotene and higher expression in tomato fruits which indicated it is the more active enzyme than that of CCD1A (Simkin et al. 2004; Ilg et al. 2014).

As the representative solanum alkaloids, steroidal glycoalkaloids (SGAs) are a series of cholesterol-derived molecules and act as dual function in tomato fruit. The most abundant of the SGAs in immature fruit, α-tomatine, is a toxic chemical to a variety of fungi, insects and human while esculeosides such as esculeogenin A in mature fruit is the health-promoting chemical which can reduce the atherogenesis (Chan Jr and Tam 1985; Fujiwara et al. 2007; Huang et al. 2015). During the ripening process, the toxic α-tomatine is transformed to the non-bitter and non-toxic esculeosides, which is catalyzed by several GLYCOALKALOID METABOLISM genes (GAMEs), such as *GAME 1/2/4/5/6/11/12/17/18/31* for the hydroxylation, acetylation and glycosylation reaction of detoxication pathway of α-tomatine (Itkin et al. 2011; Itkin et al. 2013; Alseekh et al. 2015; Cardenas et al. 2016; Cardenas et al. 2019; Szymanski et al. 2020). Moreover, based on the fine mapping method, a glycoalkaloid transporter, GORKY, has been identified which can transport α-tomatine from the store site (vacuole) to catalyzation site (cytosol) to promote the detoxication reaction (Kazachkova et al. 2021).

Moreover, as the insecticidal metabolites found in trichome in the Solanaceae, acyl-sugars are glycolipids containing two core parts: sugar cores (such as sucrose, glucose and inositol-derived disaccharide) and acyl esters chains lengths from C2 to C20 at different positions on the sugar cores (Fan et al. 2019). Based on the analysis of the isogenic introgression lines (ILs) and backcross introgression lines (BILs), three acyl-sucrose acyltransferases (ASATs) of acyl-sugar biosynthesis pathway have been cloned (Schilmiller et al. 2012; Schilmiller et al. 2015). Besides these three enzyme, another ASAT and an amino acid biosynthetic enzymes, isopropylmalate synthase like 3 (IPMS3) are also involved in the acyl-sugar biosynthesis (Ning et al. 2015; Fan et al. 2016). The detailed analysis indicates that the diversity of these genes cause the various acyl-sugar biosynthetic pathway between the different species: the truncation at the C-terminus of IPMS3 allele in *S. pennellii LA0716* results in predominant accumulation of acylsugars containing isobutyryl (isoC4), the key amino acid substitution of ASAT3 (Tyr-41-Cys) change the enzyme characteristic which cause the acyl-sugar differences between *S. lycopersicum* and *S. habrochaites* (Ning et al. 2015; Schilmiller et al. 2015).

### Regulation of fruit quality metabolism

In recent decades, after the comprehensive analysis of the enzymes that are directly involved in the metabolite pathway of fruit quality, the transcriptional, epigenetic and post-translational regulation mechanisms have become a hot topic of research (Lu et al. 2018; Wang et al. 2020).

Based on the fruitENCODE data which contains 361 transcriptome, 71 accessible chromatin, 147 histone and 45 DNA methylation profiles, Lu et al. (2018) found tomato fruit ripening is under the regulation of MADS-type transcriptional feedback circuits. As one of the most famous MADS family member, the mutant of RIN has been comprehensively investigated about its ripening-related phenomena in the past a half-century. Its fruit significantly lack the ethylene burst and as such neither changes color nor soften (Robinson 1968; Vrebalov et al. 2002; Ito et al. 2017). Although RIN may be not required for the initiation of ripening, lots of research have demonstrated that several ripening associated pathways, such as the ethylene, carotenoid, cell wall and secondary metabolism pathway, are under the regulation of RIN. The large-scale analysis of ChIP-chip and transcriptome confirmed the RIN function on ripening through the direct binding and activation of the key ripening-related structural and regulator genes, *ACS2/4*, *SGR1*, *PSY*, *Cel2*, *EXP1*, *PAL1*, *C4H*, *LoxC*, *AAT1*, *CNR*, *NOR*, *AP2a* and itself (Fujisawa et al. 2012; Fujisawa et al. 2013; Irfan et al. 2016). Moreover, as MADS-box proteins usually function with other MADSs and act as multimers to regulate certain pathways, RIN can interact with other MADS-box transcription factors (such as FUL1/2 and TAGL1) to co-regulate ripening processes (Honma and Goto 2001; Shima et al. 2013). In detail, the *TAGL1* and *FUL1*/*FUL2* knock-down mutant exhibited a significantly decreased ethylene burst and producing yellow-orange fruit with low carotenoid levels (Vrebalov et al. 2009; Shima et al. 2014; Gimenez et al. 2015). Besides the above mentioned transcription factors, recently, some novel MADS transcription factors, such as *CMB1*, *TDR4*, *MBP8* and *MBP15* have been demonstrated to act as the important regulators affecting pigmentation, secondary metabolism or cell wall metabolism further confirmed the central function of MADS transcription factors in the tomato fruit ripening process (Yin et al. 2017; Yin et al. 2018; Zhang et al. 2018a; Zhao et al. 2019).

As the fruit is developed from the floral organ and plant-specific NAC (no apical meristem (NAM), Arabidopsis transcription activator factor 1/2 (ATAF1/2) and Cup-shaped cotyledon (CUC2)) transcription factors play important roles in Arabidopsis senescence and floral development, their orthologous genes are also acting as vital regulators of fruit ripening following neofunctionalisation or repurposing of pre-existing genes (Lu et al. 2018). For example, *NAP2*, the tomato putative ortholog of *AtNAP* which is the core regulator of leaf senescence (Guo and Gan 2006), can directly regulate the gene expression of abscisic acid biosynthesis and affect the pigmentation and softening of tomato fruits (Kou et al. 2018; Ma et al. 2018). Moreover, the fine-mapping result of the non-ripening (*nor*) mutant indicated that its delayed ripening phenotype is attributed to the early termination of a NAC family TF protein translation (NOR). The truncated 186-amino-acid protein (NOR186) can compete with the wild type NOR for the accessibility to bind the promoters of *GGPPS2* and *PL* which are involved in the carotenoid biosynthesis and cell wall modification (Gao et al. 2020). Based on the systematically analysis of fruit-expressed NACs function by Virus-Induced Gene Silencing (VIGS), NOR-like1 which exhibits 62.84% amino acid homology with NOR, is identified to be involved in fruit ripening. The ripening initiation of its knock-out lines is significantly delayed by 14 days. RNA-sequencing profiling and chromatin immunoprecipitation-quantitative PCR (ChIP-qPCR) analysis further confirmed that NOR-like1 can directly bind to the promoters and activate the expression of *ACS2*, *ACS4*, *GGPPS2*, *SGR1*, *PG2a*, *PL*, *CEL2*, and *EXP1* (Gao et al. 2018b). Additionally, NAC transcription factors can also affect the ripening-related hormones biosynthesis: the knock-down fruit of *NAC4* exhibit the repression of ethylene biosynthesis and in the *NAC1*-overexpressing tomato fruit, ethylene synthesis-related genes is downregulated while the ABA biosynthesis pathway is induced (Ma et al. 2014; Zhu et al. 2014).

Although cultivated tomato fruit do-not usually accumulate anthocyanin, three loci *Anthocyanin fruit* (*Aft*), *atroviolacium* (*atv*) and *Aubergine* (*Abg*) can significantly induce the anthocyanins accumulation in cultivar fruit after the introgression from wild tomato *S. chilense*, *S. cheesmaniae* and *S. lycopersicoides*, respectively (Jones et al. 2003; Cao et al. 2017). Based on fine-mapping analysis, an R2R3-MYB transcription factor, *AN2-like* is responsible for the *Aft* phenotype and acts as an activator of anthocyanin biosynthesis. Another R3-MYB protein, *MYB-ATV* is responsible for the *atv* phenotype and can competitively interact with bHLH factors (AN1 and JAF13) of MBW complex, which acts as repressor of anthocyanin synthesis (Colanero et al. 2018). Recently, Colanero et al. (2020) reported that the alternative splicing of *AN2-like* allele represses the translation of the functional MYB protein, which finally contributes to the lack of anthocyanin pigmentation phenotype in cultivated tomato. Moreover, three other R2R3-MYB transcription factors, *ANT1*, *ANT1-like* and *AN2* are located around *Aft* loci and the overexpression of *AN2 and ANT1* can also significantly accumulate anthocyanin in cultivar tomato (Schreiber et al. 2012; Zhi et al. 2020). Besides the function on anthocyanin biosynthesis, MYB family transcription factors also act as important regulators of other metabolisms. In *AN2*-OE fruits, the expression of volatile aroma genes (*LOXC*, *AADC2* and *TPS*) are significantly induced, which attribute to the high accumulation of aroma volatiles, such as aldehyde, phenylpropanoid-derived and terpene volatiles (Jian et al. 2019). Recently, the functional characteristic of MYB72 further confirms that the MYB family can not only affect the carotenoid accumulation and chromoplast biogenesis through dual regulation of *POR*, *CHLH*, *TKN2*, *PSY*, *Z-ISO* and *LCYB* gene but also negatively regulate flavonoids and phenolic acids accumulation by repressing *4CL*, *CHS1* and *CHS2* (Wu et al. 2020).

Moreover, several other transcription factor families such as WD40 (AN11), ERFs (AP2a, ERF.B3 and ERF.G3-like), GRAS (GRAS38), ABFs (AREB1), ARFs (ARF6A, ARF4 and ARF10), BLHs (BL4), bHLHs (GL3, TT8, PRE2 and bHLH114), bZIPs (bZIP1) and HD-zip (HZ24) also play important roles in the ripening regulation processes. As the other important components of MBW complex for anthocyanin biosynthesis, AN11, a tomato WD40 protein can interact with a bHLH transcription factor TT8 corresponded to the *ah* (*Hoffman's anthocyaninless*) locus to regulate anthocyanin and flavonoid biosynthesis (Qiu et al. 2016; Gao et al. 2018a). The softening process which results from cell wall degradation is under the high order regulation of *AP2a*, *GRAS38*, *ARF4* and *BL4* transcription factors (Karlova et al. 2011; Sagar et al. 2013; Shinozaki et al. 2018; Yan et al. 2020a). Through the regulation of *PSY1*, *PDS* and *ZDS*, the fruit color of *PRE2* and *ERF.B3* transgenic fruits are dramatically changed (Liu et al. 2014; Zhu et al. 2017). Recently, based on the MicroTom Metabolic Network, two novel transcription factors, ERF.G3-like and bHLH114, are identified to be involved in the flavonoid and SGA metabolism, respectively (Li et al. 2020b).

Given that epigenetic markers such as DNA methylation affect gene expression and act as an important regulator of Arabidopsis senescence and flower development (Sung et al. 2006; Li et al. 2020c), Zhong et al. (2013) found that the methyltransferase inhibitor, 5-azacytidine can accelerate the tomato ripening process. Moreover, the fruitENCODE data indicate that the core MADS-type regulation circuit genes of tomato ripening is suppressed by the DNA hypermethylation and H3K27me3 in the promoter and gene body of the core genes at the immature fruit stage while is demethylated and activated in ripening fruit tissues (Lu et al. 2018). Tomato *Cnr* is another well-known mutant. It resulted from a spontaneous epigenetic change occurring due to the high level of methylation of a promoter causing a low expression of *CNR* (Manning et al. 2006). Similarly, in the Vitamin E pathway, the differential methylation of a SINE retrotransposon located in the promoter the causal gene, *2-methyl-6-phytylquinol methyltransferase* (*VTE3 (1)*) of mQTL9-2-6 affected its expression and then cause the variation of vitamin E among the population (Quadrana et al. 2014). Moreover, besides the repressive regulation of trimethylation of histone H3 at Lys27 (H3K27me3) (Kit et al. 2010; Boureau et al. 2016; Lu et al. 2018), histone acetylation which act as gene activator is also involved in the tomato ripening especially carotenoid biosynthesis by the activation of *histone deacetylase 3* (*HDT3*) while repression of *histone deacetylase 1/3* (*HDA1/3*) (Guo et al. 2017b; Guo et al. 2017a; Guo et al. 2018).

Post-translational regulations (such as ubiquitination, oxidation, glycosylation and phosphorylation) of the regulators and structural ripening-related proteins are also of considerable importance for fruit metabolism. The ubiquitin-proteasome system-mediated proteolysis is a crucial protein degradation pathway in eukaryotes. GLK2 which positively regulates the plastid level and the pigment accumulation, is a substrate of the CUL4-DDB1-DET1 ubiquitin ligase complex for the proteasome degradation (Tang et al. 2016). Recently, Wang et al. (2020) found that tomato PSY1 contains two ubiquitinated lysine residues and its precursor protein can interact with Plastid Protein Sensing RING E3 ligase 1 (PPSR1) to mediate its degradation via ubiquitination. Moreover, based on the iodoacetyl tandem mass tag (iodoTMT)-based redox proteomic approach, the oxidation levels of polygalacturonase 2A and 1-aminocyclopropane-1-carboxylate oxidase-like protein (E8) are significantly changed in parallel with the reactive oxygen species (ROS) fluctuate during fruit ripening, which supply novel regulation mechanisms of ROS on the of tomato ripening (Wang et al. 2021). As the glycosylation and phosphorylation are remarkable modification to produce the functional enzyme, *N*-glycosylation of tomato TIV-1 and Pectinesterase 1 is important for its enzyme activity and protein stability and the phosphorylation of sucrose synthase, hexose and CDKA directly affect the sugar metabolite and fruit development (Roessner-Tunali et al. 2003; Anguenot et al. 2006; Gonzalez et al. 2007; Tauzin et al. 2014; Zhang et al. 2020).

Although many of the major pathways and the genes involved in fruit quality related metabolite pathways have been identified, the branched pathways and the associated genes are not yet fully analyzed. In recent decades, a large amount of researches has indicated that the weighted gene correlation network analysis is a powerful method to explore the novel enzymes, potential partners in protein–protein interactions (PPIs) and regulation of aspects of metabolism (Shoemaker et al. 2007; Fukushima et al. 2012; Mandal et al. 2020). Therefore, we constructed the gene co-expression network of the presentative genes (*PSY* for carotenoid, *SGR* for chlorophyll, *PL* for cell wall, *CHS1* for secondary metabolite) based on the high-resolution spatiotemporal transcriptome data of tomato fruit development and ripening (Luo et al. 2013; Espana et al. 2014; Yang et al. 2017; Shinozaki et al. 2018; Jian et al. 2019; Segado et al. 2020; Xiong et al. 2020) (Table 2).

**Table 2:** Co-expression genes of presentative genes.

Presentative genes	Coexpressed gene	Gene name	coefficient	Presentative genes	Coexpressed gene	Gene name	coefficient
*PSY1*	Solyc05g012020	*RIN*	0.96	*CHS1*	Solyc11g013110	*FLS6*	0.93
	Solyc10g081650	*CRTISO*	0.95		Solyc10g078240	*C3H*	0.81
	Solyc12g098710	*Z-ISO*	0.83		Solyc12g088460	*F3′H-like*	0.80
	Solyc08g080090	*SGR*	0.78		Solyc01g096670	*C3′H*	0.74
	Solyc01g102950	*Lycopene beta/epsilon cyclase*	0.77		Solyc02g083860	*flavanone 3-hydroxylase*	0.91
					Solyc03g097030	*4CL*	0.74
*SGR*	Solyc12g017250	*PSBR*	0.88		Solyc03g097170	*Cinnamoyl-CoA reductase*	0.69
	Solyc12g098710	*Z-ISO*	0.83		Solyc03g115220	*F3′H*	0.94
	Solyc03g031860	*PSY*	0.78		Solyc03g117600	*HCT*	0.74
	Solyc10g081650	*CRTISO*	0.67		Solyc04g080550	*Phenylcoumaran benzylic ether reductase*	0.78
					Solyc05g052240	*CHI2*	0.87
*PL*	Solyc05g012020	*RIN*	0.884		Solyc05g053550	*CHS2*	0.92
	Solyc10g047030	*LEXYL1*	0.85		Solyc08g076790	*Cinnamoyl-CoA reductase*	0.73
	Solyc06g051800	*LeEXP1*	0.69		Solyc09g007910	*PAL5*	0.64
	Solyc09g010210	*Cel2*	0.66		Solyc09g007920	*PAL1*	0.75
	Solyc12g008840	*TBG4*	0.55		Solyc09g059170	*flavonoid glycosyltransferase genes*	0.72

Given that SGR and PSY are the important proteins involved in pigment metabolism and the former research report that SGR protein can interact with PSY, *SGR* and *PSY* are in the same co-expression network and show a high correlation (coefficient =0.78). Moreover, both of them exhibited high correlation with several gene involved in carotenoid biosynthesis, such as carotenoid isomerase (*CRTISO*, Solyc10g081650), 15-cis-ζ-carotene isomerase (*Z-ISO*, Solyc12g098710) and lycopene beta/epsilon cyclase (Solyc01g102950) (Table 1). For the cell wall metabolism, *PL* is chosen as the guide genes to construct the co-expression network. The results indicated that beta-d-xylosidase (*XYL1*, Solyc10g047030), Expansin 1(*EXP1*, Solyc06g051800), Cellulase2 (*Cel2*, Solyc09g010210) and beta-galactosidase 4 (*TBG4*, Solyc12g008840) exhibit high co-expression relation with *PL* (Table 1). Among the genes identified by the co-expression network, some of them such as *CRTISO*, *XYL1*, *EXP1*, *Cel2* and *TBG4* have previously been mentioned to be involved in tomato ripening process (Brummell et al. 1999; Isaacson et al. 2002; Smith et al. 2002; Flors et al. 2007; Zhang et al. 2018b; Li et al. 2019), which further confirms the power of the co-expression network analysis. Besides these genes, *Z-ISO* and Solyc01g102950 will be the valuable candidate genes for assessing their function in carotenoid metabolism. Furthermore, because the enzymes of each metabolism are largely located in the same site in the cell (for example SGR, PSY, CRTISO, Z-ISO and Solyc01g102950 are all located in plastid), the high co-expression of them also indicates the possibility they may interact with each other to form a complex.

As *CHS1* is the important gene catalyzed the first committed step of the multibranched flavonoid pathway, the co-expression network of *CHS1* has been constructed. The result indicates that the key gene of the multibranched pathway, such as *PAL4* (Solyc09g007920), *CHS2* (Solyc05g053550), *FLS1* (Solyc11g013110), *F3H* (Solyc02g083860), *F3′H* (Solyc03g115220) and *CHIL* (Solyc05g052240), are highly co-expressed with *CHS1* (coefficient=0.75, 0.92, 0.93, 0.91, 0.94 and 0.87, respectively). This phenomenon further confirm that phenylpropanoid pathway may be under a global regulation mechanism, such as the transcriptional regulation of MYB12 (Fernandez-Moreno et al. 2016).

## Conclusions and Future perspectives

As the identification of the genes involved in ripening related metabolic pathway is rate-limiting step to improve fruit quality, fruit researcher have paid huge of attention on it and try to accelerate it through different methods. The traditional fine-mapping strategy have identified lots of key genes of the fruit quality related metabolite change, which supply the perfect guide genes for weighted gene correlation network analysis. In future, owing to the less cost of sequencing, the combination of the traditional fine-mapping strategy, next generation sequence and new analysis methods, such as weighted gene correlation network analysis, will accelerate the identification of the novel gene to comprehensively illuminate the metabolite change and regulation mechanism of tomato fruit ripening.

For each gene, the publications which confirmed the gene function by transgenesis or genetic analysis have been listed in the table.

Pearson correlation coefficients were calculated via the function corAndPvalue from the WGCNA package (Langfelder and Horvath 2008) using the published gene expression levels of different fruit stages (Shinozaki et al. 2018).

## Data Availability

Not applicable.

## Abbreviations

PAO: Pheophorbide a monooxygenase
PPH: Pheophytin pheophorbide hydrolase
Chl: Chlorophyll
SGR: STAY-GREEN
Chl a: Chlorophyll a
Phein a: Pheophytin a
Chlide a: Chlorophyllide a
Pheide a: Pheophorbide a
RCC: Red chlorophyll catabolite
FCC: Fluorescent chlorophyll catabolite
NCCs: Nonfluorescent chlorophyll catabolites
GGPP: Geranyl geranyl pyrophosphate
PSY: Phytoene synthase
PDS: Phytoene desaturase
Z-ISO: ζ-carotene isomerase
ZDS: ζ-carotene desaturase
CRTISO: Carotenoid isomerase
LCYB: Lycopene beta cyclase
LCYE: Lycopene epsilon cyclase
BCH: β-carotene hydroxylase
ZEP: Zeaxanthin epoxidase
NSY: Neoxanthin synthase
CCD1: Carotenoid cleavage dioxygenase1
PME: Pectin methyl esterase
RG: Rhamnogalacturonase
PG: Polygalacturonase
PL: Pectate lyase
SWEET: Sugars Will Eventually be Exported Transporters
SUT: Sugar transporters
SPS: Sucrose-phosphate synthase
SS: Sucrose synthase
Ivr: Invertase
UDPG: Uridine diphosphate glucose
PEPC: Phospho*enol*pyruvate carboxylase
OAA: Oxaloacetate
CS: Citrate synthase
ALMT9: Al-ACTIVATED MALATE TRANSPORTER9
ACO-1: Aconitase-1
ICDH1: Isocitrate dehydrogenase 1
MDH: Malate dehydrogenase
PAL: Phenylalanine ammonia-lyase
C4H: Cinnamate 4-hydroxylase
4CL: 4-coumarate CoA ligase
CHS: Chalcone synthase
CHI: Chalcone isomerase
F3H: Flavanone 3-hydroxylase
DHK: Dihydrokaempferol
DHQ: Dihydroquercetin
F3′H: Flavonoid 3′-hydroxylase
FLS: Flavonol synthase
F3GlcT: Flavonoid-3-*O*-glycosyltransferase
F3'5'H: Flavonoid 3'5'-hydroxylases
DHM: Dihydromyricetin
DFR: Dihydroflavonol 4-reductase
ANS: Anthocyanin synthase
OMTs: O-methyltransferases
UFGT: UDP glucose flavonoid 3-O-glucosyl transferase
LOX: Lipoxygenase
HPL: Hydroperoxide lyase
AAT: Alcohol acyltransferase
BCAAs: Branched chain amino acids
LIS: *S*-linalool synthase
AADC2: Aromatic amino acid decarboxylase 2
GAME: GlycoAlkaloid metabolism
TPS: Terpene synthase
SGAs: Steroidal glycoalkaloids
IPMS3: Isopropylmalate synthase like 3
NAC: No apical meristem (NAM), Arabidopsis transcription activator factor 1/2 (ATAF1/2)
Aft: Anthocyanin fruit
atv: Atroviolacium
Abg: Aubergine
DAHPS: 3-deoxy-7-phosphoheptulonate synthase
HAD: Histone deacetylase
HDT: Histone deacetylase
PPSR1: Plastid Protein Sensing RING E3 ligase 1
ROS: Reactive oxygen species
PPI: Protein–protein interactions
VOCs: Volatile organic compounds
